# Percutaneous Coronary Intervention versus Optimal Medical Therapy in Patients with Chronic Total Occlusion: A Meta-Analysis

**DOI:** 10.3390/jcm13102919

**Published:** 2024-05-15

**Authors:** Sascha Macherey-Meyer, Khalid Salem, Sebastian Heyne, Max Maria Meertens, Karl Finke, Victor Mauri, Stephan Baldus, Christoph Adler, Samuel Lee

**Affiliations:** 1 Clinic III for Internal Medicine, Faculty of Medicine, University Hospital Cologne, University of Cologne, Kerpener Straße 62, 50937 Cologne, Germany; 2 Faculty of Medicine, University Hospital Cologne, University of Cologne, 50937 Köln, Germany; 3 Cardiology III—Angiology, Center of Cardiology, University Medical Center, Johannes Gutenberg-University, 55122 Mainz, Germany

**Keywords:** chronic total occlusion, CTO, percutaneous coronary intervention, chronic coronary syndrome, acute coronary syndrome, optimal medical therapy

## Abstract

**Background/Objectives:** Chronic total occlusion (CTO) is a prevalent finding in patients with coronary artery disease and is associated with increased mortality. Prior reports on the efficacy of percutaneous coronary intervention (PCI) compared to optimal medical therapy (OMT) were controversial. Following the emergence of recently published new evidence, a meta-analysis is warranted. The current meta-analysis assessed the effects of PCI compared to OMT in the treatment of CTO. **Methods:** A structured literature search was performed. Randomized controlled trials (RCTs) and non-randomized controlled studies of interventions were eligible. The primary outcome was an accumulated composite of cardiac mortality, myocardial infarction and target vessel/lesion revascularization events. **Results:** Thirty-two studies reporting on 11260 patients were included. Of these, 5712 (50.7%) were assigned to the PCI and 5548 (49.3%) were allocated to the OMT group. The primary outcome occurred in 14.6% of the PCI and 20.1% of the OMT group (12 trials, OR 0.66, 95% CI 0.50 to 0.88, *p* = 0.005, I^2^ = 67%). Subgrouping demonstrated a consistent reduction in the primary outcome for the PCI group in RCTs (six trials, OR 0.58, 95% CI 0.33 to 0.99, *p* = 0.05). The primary outcome reduction was irrespective of the study design, and it was replicable in sensitivity and subgroup analyses. Advantages in other outcomes were rather related to statistical pooling effects and dominated by observational data. **Conclusions:** CTO-PCI was associated with improved patient-oriented primary outcome compared to OMT in a study-level meta-analysis. This composite outcome effect was mainly driven by target vessel treatment, but a significant reduction in mortality and myocardial infarction was observed, irrespectively. These findings have hypothesis-generating implications. Future RCTs with adequate statistical power are eagerly awaited.

## 1. Introduction

Chronic total occlusion (CTO) is a prevalent finding affecting 15 to 33% of patients with coronary artery disease undergoing coronary angiography [1,2,3,4,5]. CTO itself is associated with increased mortality [4,6,7]. The available evidence from mainly observational data comparing percutaneous coronary intervention (PCI) to optimal medical therapy (OMT) demonstrated symptom relief, rather than a consistent reduction in major cardiovascular outcomes [8,9]. CTO-PCI is a complex coronary procedure: technical success rates depend on operators’ experience and on trial design with about 85% in dedicated CTO registries, but only 64% in national reports [10,11]. Successful CTO-PCI was associated with a more favorable outcome compared to failed PCI attempts in prior observational studies and a pooled data analysis [2,12,13,14,15,16,17]. The current European Society of Cardiology guidelines on myocardial revascularization or acute coronary syndrome (ACS) lack explicit and specific recommendations for the treatment of CTO despite a class IIa indication for revascularization in the case of refractory angina [8,18,19]. In this context, CTO-PCI mainly relies on symptomatic rather than on prognostic indication, is still a subject of discussion and requires a careful decision-making process [9,20].

Additional studies, including follow-up reports of randomized-controlled trials (RCTs, EURO-CTO [21], COMET-CTO [22]), were recently published after the latest guideline reports [8,18]. The results of these studies could potentially further inform treatment practice. Following the emergence of this new evidence, a meta-analysis is warranted, in order to further elucidate the efficacy of PCI compared to OMT in CTO treatment.

## 2. Methods

This meta-analysis was conducted using a protocol and reproducible plan for the literature search and synthesis, and it was reported according to the Preferred Reporting Items for Systematic reviews and Meta-Analysis (PRISMA) guidelines [23]. The systematic literature search was performed using three databases, including Medline (via PubMed), Web of Science, and Cochrane Library. The search strategy for each database is provided in the Supplementary Materials. The search was performed on 27 November 2023. No restrictions on the publication date, language or study size were applied. After the exclusion of duplicates and screening of titles and abstracts according to the eligibility criteria, full texts of the remaining articles were assessed. Additional handsearching of screened references was performed. The screening of titles and abstracts, full text assessment and final study selection were independently performed by two reviewers (SM, KS). In case of any disagreement, this was resolved based on a consensus with one of the senior authors (SL/CA) at each step.

Controlled trials comparing PCI to OMT in patients with CTO were included. Randomized controlled trials (RCTs) and non-randomized controlled studies of interventions (NRSIs) were eligible. In NRSI, matched data (e.g., propensity score matching, PSM) were preferred over crude data. No restrictions were applied for specific interventional or conservative treatment strategies, follow-up duration or timing of the intervention itself. Double publications, case reports, case series without control groups, reviews, meta-analyses and conference abstracts were excluded. Studies grouping revascularization techniques (e.g., PCI and coronary artery bypass grafting (CABG)) or studies with solely CABG revascularization were excluded.

Data were independently extracted by two investigators (SMM, KS) using a standardized pre-specified data collection form. In case of any disagreement, this was resolved based on a consensus with one of the senior authors (SL/CA) at each step. Main study reports, as well as any Supplementary Materials and study protocols, were reviewed. Pre-specified data elements included the study design, patient baseline characteristics, intervention and follow-up data. In the case of published follow-up reports or several reports of the same cohorts, the longest available observational period was preferred, and event data were extracted accordingly.

The primary end point was constructed as the accumulated composite equivalent to major adverse cardiac events (MACE). The primary outcome was manually calculated through the extraction of total events of cardiac mortality, myocardial infarction (MI) and target vessel revascularization (TVR) or target lesion revascularization (TLR) from the included studies. Either TVR or TLR were selected depending on available data. The primary outcome was named the *MACE equivalent*.

As TVR and TLR might be clinically less impactful, we investigated a second constructed composite called *Mortality&MI*, which consequently included cardiac mortality and MI. Only trials eligible for primary outcome analysis were considered in the *Mortality&MI* analysis to adjudicate on the robustness of the primary outcome measurement. The individual components of the primary outcome, stroke, major adverse cardiac and cerebrovascular events equivalent (MACCE, composite of primary outcome and stroke) and all-cause mortality were secondary efficacy endpoints. Peri-procedural MI was ad-hoc excluded from the analysis of MI given the uncertainty in the measurement and inequality in the distribution between the PCI and OMT group [24,25]. TVR and TLR included PCI, but CABG was also considered during the observational period.

Risk of bias (RoB) at the study level was assessed using the Cochrane Collaborations risk-of-bias tool (RoB2, version 08/22/2019) for RCTs [26]. NRSIs were assessed using the Cochrane Collaborations Risk Of Bias In Non-randomized Studies of Interventions (ROBINS-I, version 10/20/2016) tool [27].

The risk of bias assessment was independently performed by two investigators (SMM, KS). In the case of a discrepancy, a third independent investigator was consulted (SH). The risk of bias assessment was performed focusing on the primary outcome.

Random-effects meta-analyses were performed using the Mantel–Haenszel method for dichotomous event data. Pooled odds ratios (ORs) and 95% confidence intervals (CIs) are given for each analysis with a two-sided significance level of *p* < 0.05 (RevMan 5.3, Nordic Cochrane Centre, Cochrane Collaboration). The extent of heterogeneity was approximated by I^2^ tests considering 0–40% as non-important, 30–60% as moderate, 50–90% as substantial and 75–100% as considerable heterogeneity. Pre-specified funnel plot analysis was performed for the primary outcome.

A sensitivity meta-analysis of the primary outcome was performed according to the risk of bias judgement. RCTs at “high” and NRSIs with a “serious” or “critical” risk of overall bias were excluded. Subgroup meta-analysis of the primary outcome was performed according to the study design (RCT vs. NRSI), intention-to-treat principle (ITT, exclusion of studies who assigned failed PCI to OMT), patient selection (exclusion of unmatched NRSI) and underlying disease (exclusion of studies considering patients with concomitant ACS). The latter was based on the hypothesis that ACS itself might impact the MACE rate, rather than the treatment of CTO in the non-infarct related artery.

We did not obtain ethical approval for this meta-analysis because we did not collect data from individual human subjects.

## 3. Results

### 3.1. Study Selection

**Figure 1:**
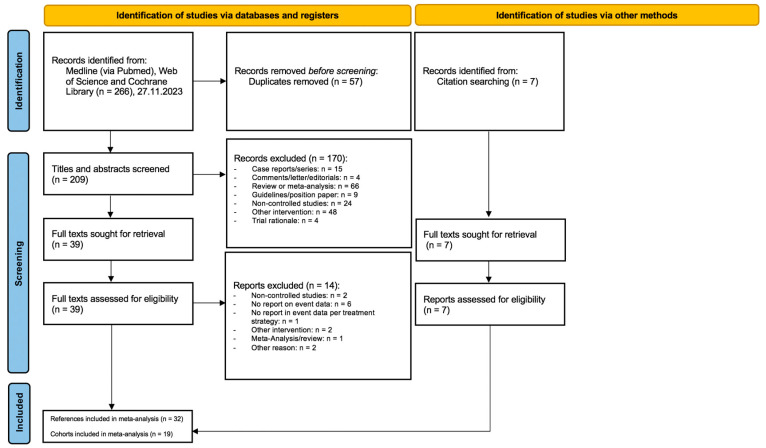
PRISMA flow chart.

A total of 266 articles were identified by the literature search (see Figure 1, PRISMA flow chart, see Supplementary Materials). After removing duplicates, the titles and abstracts of 209 remaining articles were screened. Of these, 170 articles were excluded, which left 39 references for an assessment of full-text eligibility. Seven additional full-texts were assessed for eligibility by handsearching [13,28,29,30,31,32,33]. Thirty-two publications reporting on nineteen patient cohorts were finally included in the quantitative analyses [4,13,21,22,29,30,31,32,33,34,35,36,37,38,39,40,41,42,43,44,45,46,47,48,49,50,51,52,53,54]. 

### 3.2. Studies and Cohorts

Nineteen patient cohorts were considered (see Table 1). Six cohorts were enrolled in RCTs [21,22,29,31,34], whilst the remaining were treated with NRSIs. Of these, eight were conducted using PSM [4,13,32,41,43,47,51,54], and five were case series with unmatched control groups [33,38,42,46,53]. Three RCTs [21,29,34] and two NRSIs [4,46] followed a multicentric approach. The remaining fourteen trials were monocentric studies. 

The CTO definition varied within the trials. In the majority of studies, a thrombolysis of myocardial infarction (TIMI) flow 0 with a known or estimated duration of at least or greater than 3 months was required [4,21,29,31,34,41,43,46,47,51,53,54]. The indication for CTO treatment was sparsely reported, but stabile angina pectoris combined with a proof of myocardial ischemia in the CTO territory was a recurring definition. Additionally, a few trials required myocardial viability in the CTO territory. Patients with prior CABG [4,32,41,43,46,47,51,54] or concomitant ACS [13,21,22,30,31,38,54] at the time of CTO treatment were excluded in a considerable number of cohorts. Notably, the EXPLORE trial only enrolled patients with ST-segment elevation MI, and Qin et al. exclusively included patients with ACS [32,34]. In RCTs, all patients were randomly assigned to intervention. In all but one trial [30], each patient allocated to the PCI strategy was considered in the interventional group following the ITT principle. In NRSI, the treatment decision (PCI or OMT) was at the local heart team’s discretion or followed a shared-decision making process between the treating physician and the patient. In six NRSI patients with unsuccessful PCI were allocated to the OMT arm for an efficacy analysis [13,33,41,42,46,53], and the remaining six NRSIs followed an ITT principle [4,32,38,47,51,54].

The first patient was treated in January 2002, and the last patient was treated in December 2020. The median follow-up period ranged from 4 to 56 months in the RCTs, and the corresponding interval was 1.0 to 7.9 years in NRSs.

### 3.3. Risk of Bias Assessment

The RoB assessment of RCTs is summarized in Table 2a. Two trials [22,34] were judged to be at a low risk of bias. Two other trials [21,31] were associated with “some concerns” in the overall RoB assessment. In REVASC [31], imbalances in patient characteristics and slight deviations between pre-specified and reported outcomes resulted in this judgement. In EURO-CTO [21], deviations from intended interventions with a considerable cross-over rate, especially in the PCI arm, might have affected the outcome. This reflects real-world practice but indicates some sort of bias. Two RCTs [29,30] were judged to be at high risk of bias. DECISION [29] had a remarkable cross-over rate in both directions with 19.9% of patients assigned to OMT immediately crossing over to PCI after randomization. In IMPACTOR [30], patients with unsuccessful PCI and those with non-adherence to study medication were automatically excluded from the outcome analysis. These treatment strategies each indicate a substantial risk of bias. Two trials were prematurely stopped: one [21] due to limited funding (lacking 33% of targeted patient enrollment) and one [29] because of slower enrollment than expected. This indicates a source of “other bias”, which is currently not included in the RoB2 tool, but should be acknowledged.

The risk of bias assessment of NRSIs is summarized in Table 2b. Six [4,32,43,47,51,54] NRSIs were judged to be at a moderate risk of overall bias. Six [13,38,41,42,46,53] other NRSIs were judged to be at “serious”, and one [33] was judged to be at a “critical risk” of overall bias. This judgement was primarily driven by severe imbalances between treatment groups in baseline characteristics (confounding domain). Precisely, patients in the revascularization or successful revascularization groups had more favorable baseline characteristics. In the study judged to be at a critical risk of bias, the selection into the study was very strongly related to the intervention and outcome, and the patients were extremely imbalanced in baseline characteristics without any adjusted analysis attempts [33].

Secondarily, the judgement was affected by the assignment of failed PCI patients to the OMT group (derivations from intended interventions)—which stands in contrast to higher quality trials following the ITT principle. These sources of bias might have reasonably influenced the measured outcome.

### 3.4. Patient Baseline Characteristics and Procedural Data

A total of 11,260 patients with coronary artery disease and at least one CTO were included in the analysis. Of these, 1890 (16.8%) were enrolled in RCTs and 9370 (83.2%) were included in NRSIs. Overall, 5712 (50.7%) were assigned to the PCI group and 5548 (49.3%) were allocated to the OMT group. The median/mean age ranged from 58.0 to 69.6 years, and 70.6 to 90.1% were male. An analysis of the cardiovascular risk profile demonstrated that 14.9 to 53.2% had diabetes, 6.5 to 81% had dyslipidemia, 39.9 to 91.6% had hypertension and 20.2 to 87.9% were ever smokers.

CTO lesions were predominantly located in native coronary arteries. The majority of CTO lesions were diagnosed in main vessels, including the left anterior artery descending (LAD), right coronary artery (RCA) and left circumflex artery (LCX). Only a negligible low number of patients with CTO of the left main stem or branches were considered in the studies included. RCA-CTO was documented in 40 to 78% of patients. Accordingly, RCA-CTO was the most prevalent affected vessel followed by LAD-CTO (10 to 44.8%) and LCX-CTO (10.2 to 41.7%).

The overall success rate of PCI varied from 60.2 to 100%. The mean fluoroscopy time ranged from 29.4 to 49.6 min. The contrast medium volume varied between 280 and 340.7 mL. Whenever reported, antegrade recanalization techniques (60.4 to 88%) were more commonly used than retrograde approaches, but these data were limited to RCTs. Detailed baseline, lesion and procedural characteristics are summarized in Table 1a,b.

## 4. Primary Outcome Analysis

### 4.1. MACE Equivalent

**Figure 2:**
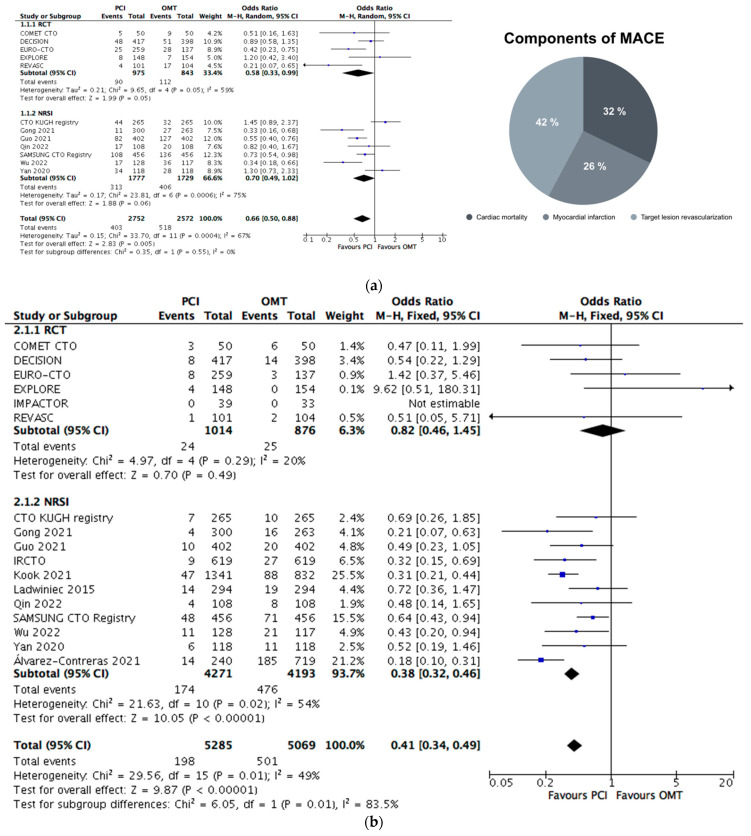

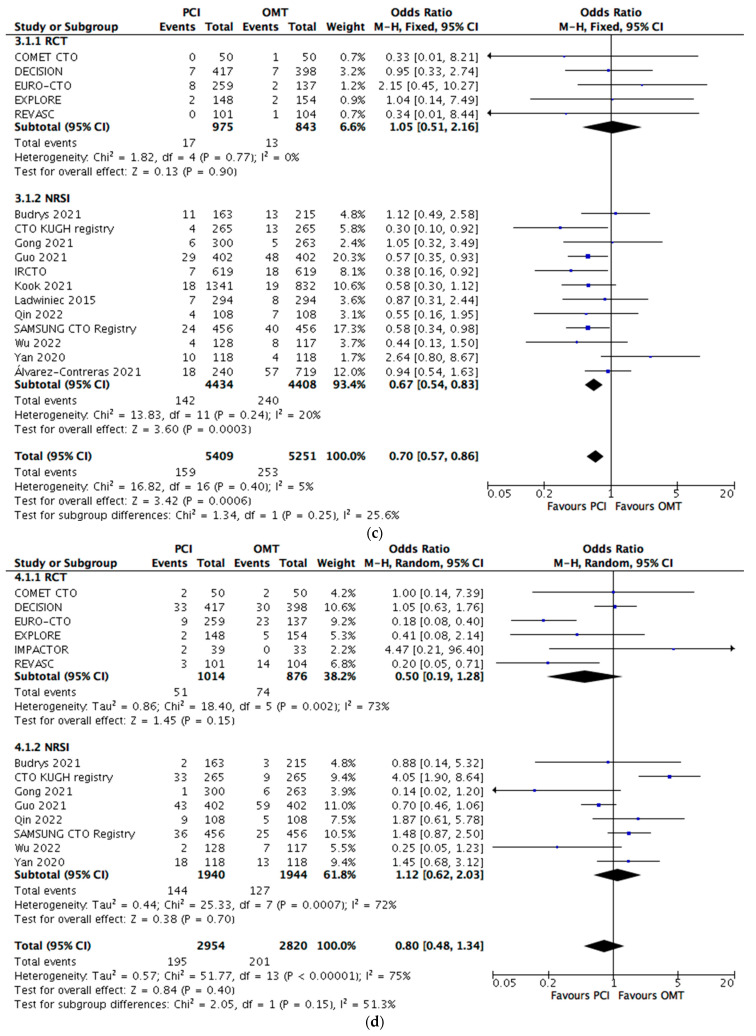

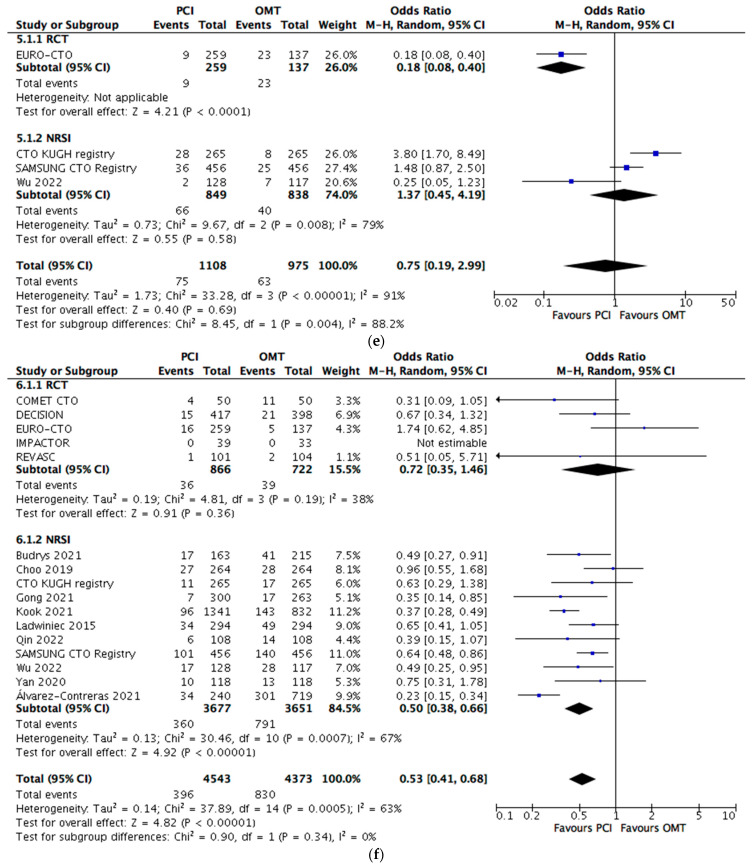
(**a**): Central illustration of primary outcome analysis. (**b**): Cardiac mortality. (**c**): Myocardial infarction. (**d**). Target vessel revascularization. (**e**): Target lesion revascularization. (**f**): All-cause mortality. Legend: OMT: optimal medical therapy, PCI: percutaneous coronary intervention, OR: odds ratio, M-H: Mantel-Haenszel method, CI: confidence interval, NRSI: non-randomized controlled studies of interventions, RCT: randomized-controlled trial.

Twelve trials were included in the analysis of the constructed primary outcome. Overall, 5324 patients were considered. Of these, 2752 were treated with PCI and 2572 were managed using OMT. The event rate was 14.6% in the PCI and 20.1% in the OMT group (see Figure 2a, OR 0.66, 95% CI 0.50 to 0.88, *p* = 0.005, I^2^ = 67%, favoring PCI, moderate heterogeneity). In detail, 296 cardiac deaths (32%), 236 MIs (26%) and 389 TVRs/TLRs (42%) were reported. In the subgroup of RCTs, 61% of events were caused by TVR/TLR, 24% of events were due to cardiac death and 15% of events were related to MI. 

A funnel plot analysis of the primary outcome did indicate slight asymmetry on the visual plot analysis (see Figure S1). Detailed events of single composite domains are summarized in Table S1.

### 4.2. Mortality&MI

All twelve trials considering 5324 patients were included in the outcome analysis. The cumulative event rate was 7.7% in the PCI and 12.4% in the OMT group (see Figure S2, OR 0.60, 95% CI 0.47 to 0.76, *p* < 0.001, I^2^ = 25%, favoring PCI, non-important heterogeneity).

### 4.3. Sensitivity Analysis of Primary Outcome

Eight trials remained for the sensitivity analysis according to the RoB assessment (see Section 3.3). The event rate was 17.2% in the PCI and 24.3% in the OMT group (see Figure S5, OR 0.66, 95% CI 0.49 to 0.90, *p* = 0.008, I^2^ = 53%, moderate heterogeneity). The observed treatment effect favoring PCI was robust in the sensitivity analysis.

### 4.4. Subgroup Analyses of Primary Outcome

Subgrouping following the trial design demonstrated a reduction in the primary outcome for the PCI group in RCTs (see Figure 2a, OR 0.58, 95% CI 0.33 to 0.99, *p* = 0.05), but this effect was not present in the NRSI comparison (OR 0.70, 95% CI 0.49 to 1.02, *p* = 0.06).

The observed treatment effect of the primary outcome reduction favoring PCI in the overall analysis was robust in the subgroup following the ITT principle (see Figure S5, OR 0.69, 95% CI 0.53 to 0.90, *p* = 0.007) and in patients with chronic coronary syndrome (OR 0.73, 95% CI 0.54 to 0.99, *p* = 0.04). But there was little to no difference after excluding unmatched trials leaving NRSIs with PSM and RCTs for analysis (see Figure S5, OR 0.75, 95% CI 0.56 to 1.00, *p* = 0.05). 

## 5. Secondary Outcome Analyses

### 5.1. Cardiac Mortality

Seventeen trials were included in the analysis of cardiac mortality. Overall, 10,354 patients were analyzed. Of these, 5285 were treated with PCI and 5069 were managed using OMT. The event rate was 3.7% in the PCI and 9.9% in the OMT group (see Figure 2b, OR 0.41, 95% CI 0.34 to 0.49, *p* < 0.001, I^2^ = 49%, favoring PCI, moderate heterogeneity).

### 5.2. Myocardial Infarction

Seventeen trials were included in the analysis of myocardial infarction. A total of 10,660 patients were assessed. Of these, 5409 were treated with PCI and 5251 were managed on OMT. The event rate was 2.9% in the PCI and 4.8% in the OMT group (see Figure 2c, OR 0.70, 95% CI 0.57 to 0.86, *p* < 0.001, I^2^ = 5%, non-relevant heterogeneity).

### 5.3. TVR

Fourteen trials were included in the analysis of TVR. Overall, 5774 patients were considered. Of these, 2954 were treated with PCI and 2820 were managed on OMT. The event rate was 6.6% in the PCI and 7.1% in the OMT group (see Figure 2d, OR 0.80, 95% CI 0.48 to 1.34, *p* = 0.40, I^2^ = 75%, substantial heterogeneity).

### 5.4. TLR

Four trials were included in the analysis of TLR. A total of 2083 patients were analyzed. Of these, 1108 were treated with PCI and 975 were managed on OMT. The event rate was 6.8% in the PCI and 6.5% in the OMT group (see Figure 2e, OR 0.75, 95% CI 0.19 to 2.99, *p* = 0.69, I^2^ = 91%, considerable heterogeneity).

### 5.5. All-Cause Mortality

Sixteen trials were included in the analysis of all-cause mortality. Overall, 8916 patients were considered. Of these, 4543 were treated with PCI and 4373 were managed on OMT. The event rate was 8.7% in the PCI and 19.0% in the OMT group (see Figure 2f, OR 0.53, 95% CI 0.41 to 0.68, *p* < 0.001, I^2^ = 63%, favoring PCI, moderate heterogeneity).

### 5.6. Stroke

Ten trials were included in the analysis of stroke. Overall, 4783 patients were considered. Of these, 2457 were treated with PCI and 2326 were managed on OMT. The event rate was 0.8% in the PCI and 1.5% in the OMT group (see Figure S3, OR 0.54, 95% CI 0.30 to 0.99, *p* = 0.05, I^2^ = 0%, favoring PCI, non-important heterogeneity).

### 5.7. MACCE equivalent

Eight trials were included in the analysis of the MACCE equivalent. A total of 3167 patients were analyzed. Of these, 1675 were treated with PCI and 1492 were managed on OMT. The event rate was 11.5% in the PCI and 16.0% in the OMT group (see Figure S4, OR 0.62, 95% CI 0.42 to 0.92, *p* = 0.02, I^2^ = 67%, favoring PCI, moderate heterogeneity).

## 6. Discussion

This meta-analysis was based on the most recent available long-term follow-up data and comprehensively assessed the effect of PCIs compared to OMT in patients with CTO. The main and novel findings of the pooled data analyses on the study-level were as follows:

▪ PCI was associated with a 34% lower likelihood of a constructed primary outcome *MACE equivalent*; this was consistent in RCTs and replicable after an adjustment for confounding factors in the ITT subgroup analysis and after the exclusion of studies considering patients with concomitant ACS.
▪ Primary outcome reduction was mainly related to TVR or TLR, but *Mortality&MI* outcome analysis PCI was associated with a decreased event rate again.
▪ PCIs were associated with a 47% decrease in all-cause mortality.
▪ PCIs were associated with a 46% lower likelihood of stroke and a subsequent 38% decrease in the MACCE equivalent.
▪ There was little to no detectable difference in TLR and TVR rates between the interventions.

Pooled data analysis is the uncontested strength of a meta-analysis, but interpretation of the overall effects requires cautious revision.

In the present context, CTO-PCI was associated with the above-mentioned advantages. In contrast, prior meta-analyses showed an improved outcome following the CTO-PCI in pooled analyses or in NRSIs but not in the RCT subgroup [55,56,57]. A funnel plot analysis indicated slight asymmetry, which could reflect publication bias and interstudy heterogeneity. Furthermore, this difference might be related to the recent long-term follow-up reports from EURO-CTO [21] and COMET-CTO [22]. Mainly, potential differences in the primary outcome definition and measurement in prior meta-analyses might be contributing factors. For example, Simsek et al. [55] did not specify the MACE definition, and Abo-Ali et al. considered MACE according to the individual study definition resulting in heterogenous reports [56]. During the extraction process, the authors of the present review identified various definitions of major adverse cardiac events used. In brief, in six RCTs we documented five different definitions:

- All cause death, MI, clinically driven repeat revascularization (REVASC, IMPACTOR);
- All cause death, MI, stroke, any revascularization (DECISION);
- Cardiac death, MI, CABG (EXPLORE);
- Cardiac death, non-fatal MI, ischemic driven TLR (EURO CTO);
- Cardiovascular death, non-fatal MI, any revascularization (COMET CTO);

In the cardiovascular literature, the definition of MACE lacks standardization; this is a major limitation in the pooled data analysis of composite outcomes [58]. Consequently, the authors defined the primary outcome *MACE equivalent* according to preexisting CTO publications in the post-hoc analysis [57]. We decided to accumulate event data of the single domains and construct a reasonable *MACE equivalent* outcome instead of considering heterogenous composites from the included studies. In doing so, the pure event rate in the present accumulated MACE analysis might be overestimated compared to conventional time-to-event composite analyses. Repeated events in the same patients might be theoretically considered, but this reflects a patient-oriented approach in the outcome assessment and evaluation. On the study-level this seems to be the most reasonable approach, but does not imply the true event rate. On the one hand, a patient-level meta-analysis might be a future approach to overcome this inconsistency. On the other hand, we evaluated each single domain of the composite to adjust for confounding factors in the data analysis.

The primary outcome difference was mainly attributable to TVR and TLR (see Figure 2a). This observation was consistent in both the overall analysis and in the subgroup of RCTs. TVR and TLR are widely accepted cardiovascular outcomes [24,59], but standardization regarding indication and interpretation in trials comparing PCI to OMT is controversial. One might assume that in the comparator group, TVR/TLR was clinically driven in patients with refractory angina pectoris despite optimized medical management. On the contrary, in the PCI group, not only a symptomatic indication but also device-failure after the PCI might have contributed to repeated revascularization. As the authors were not able to adjust for this confounding factor, we designed the *Mortalty&MI* outcome. PCI was associated with a 40% lower likelihood of MI or cardiac death in the overall analysis, but this advantage was not replicable in the subgroup of RCTs. One should bear in mind that the event rate was remarkably lower in RCTs than in NRSIs (4.3% vs.15.2%) and might lack statistical power even in the pooled data analysis [55].

Finally, the reduction in the composite of cardiac mortality, MI and TVR/TLR following PCI was a consistent measurement and was sustained in the sensitivity and subgroup analysis.

Pooled data analysis showed an approximately 50% lower likelihood of all-cause and cardiac mortality following CTO-PCI, but this was not a robust finding in the subgroup of RCTs (see Figure 2b, see Figure 2f). Firstly, single RCTs did not have enough statistical power to detect mortality differences [55]. Secondly, the advantageous mortality data were mostly related to events in NRSIs underlying especially selection bias. As previously acknowledged, patients included in CTO-RCTs have a more favorable risk profile and less complex lesions compared to real-world populations and registries [11]. The latter are at an increased risk of procedural and long-term cardiovascular complications. Accordingly, the cardiac mortality rate was 7.7% in the NRSIs and 4.2% in RCTs in the present report. Finally, the follow-up period was longer in NRSI compared to RCTs. The potential effect of PCIs on survival data might be uncertain because RCTs had this shorter follow-up period (RCTS: 4 to 56 months, NRSI: 1.0 to 7.9 years) and were not adequately powered on an individual trial level to assess survival [55,57]. Patients with CTO have an increased risk of ventricular arrhythmia [60], cardiogenic shock [61] and MI—as shown in the present analysis. CTO-PCI might optimize targeted artery and donor-vessel flow and subsequently improve overall hemodynamics and contribute to remodeling. These processes might reduce the number of events of cardiac death based on a reduction in MI, arrhythmia and shock. These hypotheses require a further work-up.

One might speculate whether PCI offers a time-dependent advantage in mortality or whether advantages in NRSIs are purely related to confounding factors. An increased follow-up of preexisting RCTs or new evidence might add further information. In this context, the authors want to point out that the present literature indicates considerably higher CTO-PCI success rates in dedicated registries by experienced operators [10,11,62,63]. The trend of successful revascularization contributing to a better prognosis might only reliably appear if patients survive the risky and complex CTO-PCI [2,12,13,14,15,16,17,64,65]. The referral of CTO patients to experienced centers might be the preferred strategy to optimize interventional and clinical outcomes in this vulnerable cohort, but this requires prospective validation [20].

The expected results from the NOBLE CTO (NCT03392415) and ISCHEMIA CTO (NCT03563417, [66]) trial, each comparing OMT to PCI in the CTO treatment, might add further evidence to the research question of optimized treatment. NOBLE CTO will enroll 2000 patients, and study completion is expected in July 2030. The primary outcome is all-cause mortality at ≥6 months. ISCHEMIA CTO will enroll 1560 patients investigating differences in major adverse cerebral and cardiovascular events after 5 years. Completion is expected in November 2032. The results of these studies could potentially further inform treatment practice and are eagerly awaited. Until then, angina relief and quality of life improvement remain the leading indication for CTO-PCI [14,18,19,20,67].

### Limitations and Strengths

Confounders on the individual study level and interstudy heterogeneity were acknowledged. The intervention itself varied throughout the trials, and this restricts generalizability: the majority of patients were treated in dedicated CTO-PCI centers with specific interventional expertise, most trials were single-center reports, the treatment decision had substantial confounding potential in NRSIs and the indication for treatment varied. The single-blind design might have contributed to cross-over rates in RCTs with up to one-fifth in DECISION. Uncertainty of the treatment decision regarding TVR/TLR, especially in OMT groups in primary studies, are an inherent limitation. The heterogeneity in follow-up limits transferability, especially in the survival analysis. Both subgroup and sensitivity analyses were performed to adjust for these sources of selection, detection and performance bias. As mentioned before, the primary outcome construction might overrate the measured effect of CTO-PCI, but this follows a patient-oriented approach.

The extracted data were restricted to study-level reports, and the authors did not have access to patient-level data. The latter would have allowed for a more accurate analysis of patient- and procedural-related differences (e.g., collateralization vs. insufficient collateralization in CTO territory, calcification vs. no calcification, treatment regimen, stent type, intravascular imaging, antegrade vs. retrograde approach, lesion crossing technique, successful vs. failed PCI). Procedural complications were inconsistently reported and not analyzed in this review. Accordingly, type 4a myocardial infarction was excluded from the analysis of MI [68]. This might have favored the outcome of the PCI group. The authors faced an unclear overlap of two patient cohorts [46,51], and even a detailed revision of the manuscripts and supplements did not result in clarification. Accordingly, only one trial [51] was considered in the primary outcome assessment to overcome this inconsistency. Moreover, the explicit combination of OMT and adherence to contemporary guidelines could not be assessed on the trial level.

The important strengths of this meta-analysis are the systematic description and discussion of bias, the demonstration of weaknesses and gaps in the evidence and the adjusted analysis of most recent available long-term data.

## 7. Conclusions

CTO-PCI was associated with an advantageous patient-oriented primary outcome—a composite of accumulated event rates of cardiac mortality, MI, TVR/TLR—compared to OMT in the study-level meta-analysis. This benefit was mainly driven by TVR/TLR. The primary outcome reduction was replicable in sensitivity and subgroup analysis of the RCTs or following the ITT principle.

Cardiac mortality, all-cause mortality, MI and stroke were decreased in the CTO-PCI group, but this was mainly driven by pooling effects dominated by observational data. Deaths were less frequently observed in RCTs, and the authors assume a lack of statistical power in assessment of this outcome. These results have hypothesis-generating implications. Future RCTs with adequate statistical power are eagerly awaited to clarify the optimal treatment strategy in CTO.

## 8. Impact on Daily Practice

CTO-PCI was superior to OMT in the pooled data analysis measuring a patient-oriented composite outcome of cardiac mortality, myocardial infarction and revascularization of the target vessel. This hypothesis-generating finding arises from considerable bias and requires confirmation based on large scaled randomized controlled studies. Until then, these data might contribute to the decision-making process from the interventionalist’s perspective.

## Figures and Tables

**Table 1:** Characteristics of the included studies and patients. (a) Randomized-controlled trials; (b) non-randomized controlled studies of interventions. Legend: ACS: acute coronary syndrome; CABG; Coronary artery bypass grafting; CS: case series; PSM: propensity score matching; RCT: randomized controlled trial; OMT: optimal medical therapy, CTO: chronic total occlusion, PCI: percutaneous coronary intervention, TIMI: thrombolysis in myocardial infarction, LVEF: left ventricular ejection fraction; LAD: left anterior descending artery, RCA: right coronary artery, LCX: left circumflex artery, MVD: multi-vessel disease; -: not explicitly reported []: standard deviation; (): percentages; ‡: mean instead of median; †: median instead of mean; #: missing data considered.

**(a) Randomized-controlled trials**																
**Trials**				**EXPLORE**		**REVASC**		**IMPACTOR**			**DECISION**		**EURO-CTO**		**COMET-CTO**	
**Study characteristics**																
**Study period**				11/2007–04/2015		08/2007–09/2015		10/2010–04/2014			03/2010–09/2016		03/2012–05/2015		10/2015–05/2017	
**Study design**				RCT, multicentric, Europe and North America, 1:1 randomization		RCT, monocentric, Germany, 1:1 randomization		RCT, monocentric, Russia, 1:1 randomization			RCT, multicentric, Asia, 1:1 randomization		RCT, multicentric, Europe. 2:1 randomization		RCT, monocentric, Serbia, 1:1 randomization	
**Randomized patients**				304, 302 analyzed		205		94, 72 analyzed			834, 815 analyzed		448, 396 analyzed		100, 99 analyzed	
**CTO definition**				TIMI 0, vessel diameter ≥2.5 mm		TIMI 0 ≥3 months, vessel diameter 2.5 to 4 mm		-			TIMI 0 ≥3 months, vessel diameter ≥2.5 mm		TIMI 0 ≥3 months, vessel diameter ≥2.5 mm		TIMI 0, vessel diameter ≥2.5 mm	
**Indication for treatment**				CTO as bystander in ACS		Stable angina, proof of myocardial ischemia and myocardial viability		Stable angina, proof of myocardial ischemia			-		Symptomatic lesion, proof of myocardial viability		Stable angina, proof of myocardial ischemia and myocardial viability	
**Exclusion of prior CABG**				No		No		No			No		No		No	
**Exclusion of patients with concomitant ACS**				No		Yes		Yes			Yes (ST segment myocardial infarction)		Yes		Yes	
**Inclusion of failed revascularization in PCI group** Intention-to-treat principle				Yes		Yes		No			Yes		Yes		Yes	
**Comparator treatment**				OMT		OMT		OMT			OMT		OMT		OMT	
**Follow-up period, median**				4 months		12 months		12 months			48 months		37.4 months ‡		56 months ‡	
**Baseline characteristics of patients included**																
**PCI**		*Patients, n*		148		101		39			417		259		50	
**OMT**		*Patients, n*		154		104		33			398		137		50	
**PCI**		*Age, median*		60 [10]		65		-			62.2 [10.2] ‡		65.2 [9.7]		61 (7) ‡	
**OMT**		*Age, median*		60 [10]		68		-			62.9 [9.9] ‡		64.7 [9.9]		63 (5) ‡	
**PCI**		*Male patients, n*		131 (88.5)		91 (90.1)		-			344 (83.3) #		215 (83.0)		38 (76)	
**OMT**		*Male patients, n*		126 (81.8)		90 (86.5)		-			319 (81.6) #		118 (86.1)		44 (88)	
**PCI**		*Hypertension, n*		59 (39.9)		81 (80.2)		-			262 (63.4) #		189 (73.0)		43 (86)	
**OMT**		*Hypertension, n*		69 (44.8)		93 (89.4)		-			238 (60.9) #		98 (71.5)		43 (86)	
**PCI**		*Diabetes, n*		22 (14.9)		32 (31.6)		-			132 (32.0) #		85 (32.8)		14 (28)	
**OMT**		*Diabetes, n*		25 (16.2)		31 (29.8)		-			134 (34.3) #		40 (29.2)		18 (36)	
**PCI**		*Smoking, n*		77 (52.0)		23 (22.8)		-			125 (30.3) #		190 (73.4)		30 (60)	
**OMT**		*Smoking, n*		76 (49.4)		21 (20.2)		-			102 (26.1) #		92 (67.2)		37 (74)	
**PCI**		*Dyslipidemia, n*		51 (34.5)		-		-			249 (60.3) #		210 (81.1)		36 (72)	
**OMT**		*Dyslipidemia, n*		52 (33.8)		-		-			217 (55.5) #		111 (81.0)		35 (70)	
**PCI**		*LVEF, mean*		41 [11] †		-		-			57.3 [9.8] #		54.5 [10.8]		54.9 [9.4]	
**OMT**		*LVEF, mean*		42 [12] †		-		-			57.6 [9.1] #		55.7 [10.8]		51.3 [11.3]	
**PCI**		*MVD, n*		-		87 (86.1)		-			302 (73.1)		129 (49.8)		-	
**OMT**		*MVD, n*		-		94 (90.4)		-			288 (73.7)		75 (54.7)		-	
**PCI**		*CTO location RCA, n*		64 (43.2)		58 (57.4)		39/39 (100)			186 (45.0)		165/259 (63.7)		28 (56)	
**OMT**		*CTO location RCA, n*		78 (50.6)		71 (68.3)		33/33 (100)			186 (47.6)		81/141 (57.4)		39 (78)	
**PCI**		*CTO location LAD, n*		36 (24.3)		23 (22.8)		0 (0)			185 (44.8)		66/259 (25.5)		12 (24)	
**OMT**		*CTO location LAD, n*		39 (25.3)		17 (16.3)		0 (0)			163 (41.7)		38/141 (27.0)		5 (10)	
**PCI**		*CTO location LCX, n*		48 (32.4)		20 (19.8)		0 (0)			42 (10.2)		28/259 (10.8)		10 (20)	
**OMT**		*CTO location LCX, n*		37 (24.0)		16 (15.4)		0 (0)			42 (10.7)		22/141 (15.6)		6 (12)	
**PCI**		*CTO location other, n*		0 (0)		0 (0)		0 (0)			0 (0)		0 (0)		0 (0)	
**OMT**		*CTO location other, n*		0 (0)		0 (0)		0 (0)			0 (0)		0 (0)		0 (0)	
**PCI**		*J-CTO score, median*		2 [1]		2		-			2.1 [1.2] ‡		1.82 [1.07] ‡		1.48 [1.27] ‡	
**OMT**		*J-CTO score, median*		2 [1]		2		-			2.2 [1.2] ‡		1.67 [0.91] ‡		1.72 [1.09] ‡	
**Procedural aspects in PCI group**																
*Number of PCI procedures/attempts*				147		-					384		274		50	
*Successful CTO-PCI, n*				106 (72.1)		89/101 (88.1)		39/39 (100)			348/384 (90.6)		220/254 (86.6)		47/50 (94)	
*Antegrade approach, n*				124 (84.3)		61/101 (60.4)		-			-		176/274 (64.2)		44(50 (88)	
*Retrograde approach, n*				23 (15.7)		40/101 (39.6)		-			-		98/274 (35.8)		3/50 (6)	
*Fluoroscopy time, (min), mean*				-		37 †		-			42 [34.0]		49.6 [34.9]		29.4 [21]	
*Contrast volume (mL), mean*				-		280 †		-			340.7 [156.9]		285 [198]		289.6 [105.6]	
**(b) Non-randomized controlled studies of interventions**																
**Trials**				**Álvarez-Contreras 2021** [33]		**Budrys 2021** [38]			**CTO KUGH registry 2018**		**Choo 2019** [41]		**Gong 2021** [42]		**Guo 2021** [43]	
**Study characteristics**																
**Study period**				2010–2014		06/2014–12/2018			01/2004–11/2015		01/2004–12/2010		06/2017–10/2019		01/2007–12/2018	
**Study design**				Retrospective CS, monocentric, Spain		Retrospective CS, monocentric, Lithuania			Retrospective CS, PSM, monocentric, South Korea		Retrospective CS, PSM, monocentric, South Korea		Retrospective CS, monocentric, China		Retrospective CS, PSM, monocentric, China	
**Included patients**				959		378			316		528		563		804	
**CTO definition**				TIMI 0, >3 months		-			TIMI 0, vessel diameter >2.5 mm		TIMI 0, >3 months		TIMI 0, ≥3 months		TIMI 0, ≥3 months	
**Indication for treatment**				-		-			-		-		Stable Angina, acute coronary syndrome		-	
**Exclusion of prior CABG**				No		No			.		Yes		No		Yes	
**Exclusion of patients with concomitant ACS**				No		Yes			Yes		No		No		Yes	
**Inclusion of failed revascularization in PCI group** Intention-to-treat principle				No		Yes			No		No		No		Yes	
**Comparator treatment**				OMT		OMT			OMT		OMT		OMT		OMT	
**Follow-up period, median**				4.3 years		3.6 years ‡			4 years ‡		2.2 years		1 year		2.6 years	
**Baseline characteristics of patients included**																
**PCI**			*Patients, n*	240		163			265		264		300		402	
**OMT**			*Patients, n*	719		215			265		264		263		402	
**PCI**			*Age, median*	62.8 [10.8] ‡		65.9 [11.3] ‡			64.2 [9.8]		61.5 [9.8] ‡		63.0 [10]		-	
**OMT**			*Age, median*	69.6 [10.8] ‡		69.2 [9.4] ‡			64.5 [10.3]		61.5 [10.5] ‡		67.0 [11]		-	
**PCI**			*Male patients, n*	203 (84.6)		115 (70.6)			198 (74.7)		199 (75.4)		248 (82.7)		-	
**OMT**			*Male patients, n*	595 (82.8)		165 (76.7)			195 (73.5)		201 (76.1)		208 (79.1)		-	
**PCI**			*Hypertension, n*	163 (67.9)		148 (90.8)			170 (64.1)		156 (59.1)		220 (73.3)		-	
**OMT**			*Hypertension, n*	547 (76.1)		197 (91.6)			166 (62.6)		159 (60.2)		193 (73.4)		-	
**PCI**			*Diabetes, n*	79 (32.9)		51 (31.3)			113 (42.6)		117 (44.3)		119 (39.7)		-	
**OMT**			*Diabetes, n*	323 (44.9)		48 (22.3)			119 (44.9)		121 (45.8)		140 (53.2)		-	
**PCI**			*Smoking, n*	49 (20.4)		-			136 (51.3)		71 (26.9)		200 (66.7)		-	
**OMT**			*Smoking, n*	239 (33.2)		-			146 (55.0)		64 (24.2)		171 (65.0)		-	
**PCI**			*Dyslipidemia, n*	156 (65.0)		-			83 (31.3)		-		162 (54.0)		-	
**OMT**			*Dyslipidemia, n*	486 (67.6)		-			81 (30.5)		-		157 (59.7)		-	
**PCI**			*LVEF, mean*	49 [13]		-			51.0 [11.9]		54.8 [11.3]		61 [10]		-	
**OMT**			*LVEF, mean*	44 [14]		-			49.2 [12.6]		53.6 [12.3]		58 [11]		-	
**PCI**			*MVD, n*	180 (75.0)		-			188 (70.9)		-		-		-	
**OMT**			*MVD, n*	581 (80.8)		-			195 (73.5)		-		-		-	
**PCI**			*CTO location RCA, n*	106 (44.2)		-			118 (44.5)		124 (47.0)		-		-	
**OMT**			*CTO location RCA, n*	370 (51.5)		-			123 (46.4)		118 (44.7)		-		-	
**PCI**			*CTO location LAD, n*	65 (27.1)		-			79 (29.8)		84 (31.8)		-		-	
**OMT**			*CTO location LAD, n*	137 (19.1)		-			81 (30.5)		80 (30.3)		-		-	
**PCI**			*CTO location LCX, n*	43 (17.9)		-			92 (34.7)		66 (25.0)		-		-	
**OMT**			*CTO location LCX, n*	132 (18.4)		-			81 (30.5)		72 (27.3)		-		-	
**PCI**			*CTO location other, n*	27 (11.3)		-			2 (0.7)		0 (0)		-		-	
**OMT**			*CTO location other, n*	80 (11.1)		-			1 (0.3)		0 (0)		-		-	
**PCI**			*J-CTO score, median*	-		-			-		-		-		-	
**OMT**			*J-CTO score, median*	-		-			-		-		-		-	
**Procedural aspects in PCI group**																
*Number of PCI procedures/attempts*				-		-			265		-		-		-	
*Successful CTO-PCI, n*				-		143 (87.7)			265 (100)		-		-		-	
*Antegrade approach, n*				-		-			-		-		-		-	
*Retrograde approach, n*				-		-			-		-		-		-	
*Fluoroscopy time, (min), mean*				-		-			-		-		-		-	
*Contrast volume (mL), mean*				-		-			-		-		-		-	
**(1b) Non-randomized controlled studies of interventions continued**																
**Trials**				**Kook 2021** [46]	**Ladwiniec 2015** [47]		**SAMSUNG CTO registry**			**IRCTO, Tomasello**		**Qin 2022** [32]		**Wu 2022** [53]		**Yan 2020** [54]
**Study characteristics**																
**Study period**				03/2008–12/2014	01/2002–12/2007		03/2003–02/2012			03/2008–03/2009		07/2011–07/2019		01/2012–12/2020		01/2007–12/2017
**Study design**				Retrospective CS, three centers, South Korea	Retrospective CS, PSM, monocentric, United Kingdom		Retrospective CS, PSM, monocentric, South Korea, Samsung Medical Center			Retrospective CS, PSM, multicentric, Italy		Retrospective CS, PSM, monocentric, China		Retrospective CS, monocentric, China		Retrospective CS, PSM, monocentric, China
**Included patients**				2173	588		912			1238		216		261		236
**CTO definition**				TIMI 0, >3 months	TIMI 0, ≥3 months		TIMI 0, >3 months			TIMI 0, >3 months		TIMI 0, ≥3 months, ≥2.5 mm		TIMI 0, ≥3 months,		TIMI 0, ≥3 months
**Indication for treatment**				Angina, proof of myocardial ischemia	-		Angina, proof of myocardial ischemia			-		Acute coronary syndrome		Angina Myocardium viability		Angina, silent ischemia
**Exclusion of prior CABG**				Yes	Yes		Yes			Yes		Yes		No		Yes
**Exclusion of patients with concomitant ACS**				-	No		Yes (ST-segment elevation myocardial infarction)			No		No		-		Yes
**Inclusion of failed revascularization in PCI group** Intention-to-treat principle				No	Yes		Yes			Yes		Yes		No		Yes
**Comparator treatment**				OMT	OMT		OMT			OMT		OMT		OMT		OMT
**Follow-up period, median**				1138 days	5 years		7.9 years			1 year		946 days		38 months		45 months
**Baseline characteristics of patients included**																
**PCI**	*Patients, n*			1341	294		456			619		108		135		118
**OMT**	*Patients, n*			832	294		456			619		108		126		118
**PCI**	*Age, median*			-	64.3 [10.0] ‡		64.6 [10.1] ‡			68.1 [10.3] ‡		61.7 [12.4] ‡		64.8 [9.0] ‡		62.0
**OMT**	*Age, median*			-	63.9 [10.2] ‡		64.6 [11.7] ‡			68.5 [12.5] ‡		62.5 [12.0] ‡		68.4 [9.6] ‡		58.0
**PCI**	*Male patients, n*			-	220 (74.8)		370 (81.1)			515 (83.2)		97 (89.8)		105 (77.8)		90 (76.3)
**OMT**	*Male patients, n*			-	220 (74.8)		354 (77.6)			525 (84.8)		97 (89.8)		95 (75.4)		93 (78.8)
**PCI**	*Hypertension, n*			-	159 (54.1)		302 (66.2)			489 (79.0)		73 (67.6)		91 (67.4)		84 (71.2)
**OMT**	*Hypertension, n*			-	157 (53.4)		301 (66.0)			486 (78.5)		74 (68.5)		80 (63.5)		79 (66.9)
**PCI**	*Diabetes, n*			-	62 (21.1)		218 (47.8)			194 (31.3)		37 (34.3)		51 (37.8)		-
**OMT**	*Diabetes, n*			-	53 (18.0)		211 (46.3)			182 (29.4)		35 (32.4)		37 (42.5)		-
**PCI**	*Smoking, n*			-	198 (67.4)		141 (30.9)			264 (42.6)		40 (37.0)		98 (72.6)		68 (57.6)
**OMT**	*Smoking, n*			-	203 (69.1)		131 (28.7)			274 (44.3)		40 (37.0)		84 (87.9)		66 (55.9)
**PCI**	*Dyslipidemia, n*			-	146 (49.7)		113 (24.8)			390 (63.0)		12 (11.1)		-		59 (50.0)
**OMT**	*Dyslipidemia, n*			-	135 (45.9)		115 (25.2)			390 (63.0)		7 (6.5)		-		61 (51.7)
**PCI**	*LVEF, mean*			-	-		55.5 [11.7]			-		53.4 [9.5]		30.9 [6.9]		62.0 †
**OMT**	*LVEF, mean*			-	-		55.4 [11.7]			-		52.3 [10.3]		31.4 [6.6]		61.5 †
**PCI**	*MVD, n*			-	158 (53.8)		360 (78.9)			-		-		121 (89.6)		-
**OMT**	*MVD, n*			-	150 (51.1)		360 (78.9)			-		-		116 (92.1)		-
**PCI**	*CTO location RCA, n*			-	-		239 (52.4)			-		47 (43.5)		54 (40.0)		54 (45.8)
**OMT**	*CTO location RCA, n*			-	-		239 (52.4)			-		47 (43.5)		51 (40.5)		49 (41.5)
**PCI**	*CTO location LAD, n*			-	-		132 (28.9)			-		21 (19.4)		51 (37.8)		33 (28.0)
**OMT**	*CTO location LAD, n*			-	-		132 (28.9)			-		17 (15.7)		46 (36.5)		36 (30.5)
**PCI**	*CTO location LCX, n*			-	-		157 (34.4)			-		40 (37.0)		30 (22.2)		31 (26.3)
**OMT**	*CTO location LCX, n*			-	-		158 (34.6)			-		45 (41.7)		29 (23.0)		33 (28.0)
**PCI**	*CTO location other, n*			-	-		0 (0)			-		0 (0)		0 (0)		0 (0)
**OMT**	*CTO location other, n*			-	-		0 (0)			-		0 (0)		0 (0)		0 (0)
**PCI**	*J-CTO score, median*			-	-		-			-		-		2.5 [0.9] ‡		-
**OMT**	*J-CTO score, median*			-	-		-			-		-		2.7 [0.8] ‡		-
**Procedural aspects in PCI group**																
*Number of PCI procedures/attempts*				-	294		-			-		-		172		-
*Successful CTO-PCI, n*				-	177 (60.2)		-			-		-		135 (78.5)		-
*Antegrade approach, n*				-	-		-			-		-		-		-
*Retrograde approach, n*				-	-		-			-		-		-		-
*Fluoroscopy time, (min), mean*				-	-		-			-		-		-		-
*Contrast volume (mL), mean*				-	-		-			-		-		-		-

**Table 2:** Risk of bias assessment of included studies. (a) Risk of bias assessment of randomized controlled trials; (b) risk of bias assessment of non-randomized controlled studies of interventions.

(a)														
Risk of Bias	Randomization			Deviations from Intended Interventions			Missing Outcome Data			Measurement of the Outcomes			Selection of the Reported Results	Overall Risk of Bias
**EXPLORE**	Low risk			Low risk			Low risk			Low risk			Low risk	Low risk
**REVASC**	Some concerns			Low risk			Low risk			Low risk			Some concerns	Some concerns
**IMPACTOR**	Some concerns			High risk			Some concerns			Some concerns			Some concerns	High risk
**DECISION**	Low risk			High risk			Low risk			Low risk			Some concerns	High risk
**EURO-CTO**	Low risk			Some concerns			Some concerns			Low risk			Low risk	Some concerns
**COMET-CTO**	Low risk			Low risk			Low risk			Low risk			Low risk	Low risk
**(b)**														
**Risk of Bias**	**Confounding**		**Patient Selection**		**Classification of Intervention**		**Deviations from the Intended Interventions**		**Missing Data**		**Outcome Measurement**		**Selection of the Reported Results**	**Overall Risk of Bias**
**Álvarez-Contreras 2021** [33]	Critical		Moderate		Moderate		Serious		Moderate		Low		Moderate	Critical
**Budrys 2021** [38]	Serious		Moderate		Low		Low		Low		Low		Moderate	Serious
**CTO KUGH registry 2018**	Moderate		Moderate		Moderate		Serious		Low		Low		Moderate	Serious
**Choo 2019** [41]	Moderate		Moderate		Low		Serious		Low		Low		Moderate	Serious
**Gong 2021** [42]	Serious		Moderate		Moderate		Serious		Moderate		Low		Moderate	Serious
**Guo 2021** [43]	Moderate		Moderate		Low		Low		Low		Low		Moderate	Moderate
**Kook 2021** [46]	Serious		Moderate		Moderate		Serious		Low		Low		Moderate	Serious
**Ladwiniec 2015** [47]	Moderate		Moderate		Low		Low		Low		Low		Moderate	Moderate
**SAMSUNG CTO registry**	Moderate		Moderate		Low		Low		Moderate		Low		Moderate	Moderate
**IRCTO**	Moderate		Moderate		Low		Low		Low		Low		Moderate	Moderate
**Qin 2022** [32]	Moderate		Moderate		Low		Low		Low		Low		Moderate	Moderate
**Wu 2022** [53]	Serious		Moderate		Moderate		Serious		Moderate		Low		Moderate	Serious
**Yan 2020** [54]	Moderate		Moderate		Low		Low		Low		Low		Moderate	Moderate

## Data Availability

Data are available and can be extracted from the studies included.

## Supplementary Materials

The following supporting information can be downloaded at: https://www.mdpi.com/article/10.3390/jcm13102919/s1, Figure S1: Funnel plot of primary outcome analysis. Legend: Funnel plot showing odds ratios for MACE on the *x*-axis and standard errors on the y-axis; Figure S2: Mortality&MI. Legend: OMT: optimal medical therapy, PCI: percutaneous coronary intervention, OR: odds ratio, M-H: Mantel–Haenszel method, CI: confidence interval; Figure S3: Stroke. Legend: OMT: optimal medical therapy, PCI: percutaneous coronary intervention, OR: odds ratio, M–H: Mantel-Haenszel method, CI: confidence interval; Figure S4: Major adverse cardiac and cerebrovascular events equivalent. Legend: OMT: optimal medical therapy, PCI: percutaneous coronary intervention, OR: odds ratio, M-H: Mantel–Haenszel method, CI: confidence interval; Figure S5: Multipaneled figure of sensitivity and subgroup analyses. A: Subgroup analysis: intention-to-treat principle, B: subgroup analysis: exclusion of unmatched trials, C: subgroup analysis: exclusion of studies considering concomitant acute coronary syndrome, D: sensitivity analysis: according to risk of bias assessment. OMT: optimal medical therapy, PCI: percutaneous coronary intervention, OR: odds ratio, M-H: Mantel–Haenszel method, CI: confidence interval; Table S1: Event data on efficacy outcomes. Legend: OMT: optimal medical therapy, PCI: percutaneous coronary intervention.

## Abbreviations

CABG	Coronary artery bypass graft
CTO	Chronic total occlusion
CI	Confidence interval
ITT	Intention-to-treat
LAD	Left anterior artery descending
LCX	Left circumflex artery
LVEF	Left ventricular ejection fraction
MI	Myocardial infarction
MVD	Multi-vessel disease
NRSI	Non-randomized controlled study of intervention
OMT	Optimal medical treatment
OR	Odds ratio
PCI	Percutaneous coronary intervention
PSM	Propensity-score matching
RCA	Right coronary artery
RCT	Randomized controlled trial
RoB	Risk of bias
TIMI	Thrombolysis in myocardial infarction
TLR	Target lesion revascularization
TVR	Target vessel revascularization
