# Supplementary material for: Percutaneous Coronary Intervention versus Optimal Medical Therapy in Patients with Chronic Total Occlusion: A Meta-Analysis
Source: J Clin Med. 2024 May 15;13(10):2919. doi: 10.3390/jcm13102919 (PMC11122436; doi:10.3390/jcm13102919)
Supplement: Supplementary file 1 [file jcm-13-02919-s001.zip › jcm-2988688-supplementary.pdf]

Search details

Medline via Pubmed

(<chronic total occlusion> OR <CTO> OR <chronic coronary occlusion>) AND (<percutaneous coronary intervention> OR <PCI>) AND (<optimal medical treatment> OR <conservative treatment>)

((("chronic"[All Fields] OR "chronical"[All Fields] OR "chronically"[All Fields] OR "chronicities"[All Fields] OR "chronicity"[All Fields] OR "chronicization"[All Fields] OR "chronics"[All Fields]) AND ("total"[All Fields] OR "totaled"[All Fields] OR "totaling"[All Fields] OR "totalled"[All Fields] OR "totalling"[All Fields] OR "totals"[All Fields]) AND ("dental occlusion"[MeSH Terms] OR ("dental"[All Fields] AND "occlusion"[All Fields]) OR "dental occlusion"[All Fields] OR "occlusion"[All Fields] OR "occlused"[All Fields] OR "occlusions"[All Fields] OR "occlusive"[All Fields] OR "occlusives"[All Fields])) OR ("gms curr top otorhinolaryngol head neck surg"[Journal] OR "cells tissues organs"[Journal] OR "cardiovasc thorac open"[Journal] OR "cto"[All Fields]) OR (("chronic"[All Fields] OR "chronical"[All Fields] OR "chronically"[All Fields] OR "chronicities"[All Fields] OR "chronicity"[All Fields] OR "chronicization"[All Fields] OR "chronics"[All Fields]) AND ("coronary occlusion"[MeSH Terms] OR ("coronary"[All Fields] AND "occlusion"[All Fields]) OR "coronary occlusion"[All Fields]))) AND ("percutaneous coronary intervention"[MeSH Terms] OR ("percutaneous"[All Fields] AND "coronary"[All Fields] AND "intervention"[All Fields]) OR "percutaneous coronary intervention"[All Fields] OR "PCI"[All Fields]) AND ((("optimal"[All Fields] OR "optimality"[All Fields] OR "optimally"[All Fields] OR "optimization"[All Fields] OR "optimizations"[All Fields] OR "optimize"[All Fields] OR "optimized"[All Fields] OR "optimizer"[All Fields] OR "optimizers"[All Fields] OR "optimizes"[All Fields] OR "optimizing"[All Fields]) AND ("medic"[All Fields] OR "medical"[All Fields] OR "medicalization"[MeSH Terms] OR "medicalization"[All Fields] OR "medicalizations"[All Fields] OR "medicalize"[All Fields] OR "medicalized"[All Fields] OR "medicalizes"[All Fields] OR "medicalizing"[All Fields] OR "medically"[All Fields] OR "medicals"[All Fields] OR "medicated"[All Fields] OR "medication s"[All Fields] OR "medics"[All Fields] OR "pharmaceutical preparations"[MeSH Terms] OR ("pharmaceutical"[All Fields] AND "preparations"[All Fields]) OR "pharmaceutical preparations"[All Fields] OR "medication"[All Fields] OR "medications"[All Fields]) AND ("therapeutics"[MeSH Terms] OR "therapeutics"[All Fields] OR "treatments"[All Fields] OR "therapy"[MeSH Subheading] OR "therapy"[All Fields] OR "treatment"[All Fields] OR "treatment s"[All Fields])) OR ("conservative treatment"[MeSH Terms] OR ("conservative"[All Fields] AND "treatment"[All Fields]) OR "conservative treatment"[All Fields]))

Translations

chronic: *"chronic"[All Fields] OR "chronical"[All Fields] OR "chronically"[All Fields] OR "chronicities"[All Fields] OR "chronicity"[All Fields] OR "chronicization"[All Fields] OR "chronics"[All Fields]*

total: *"total"[All Fields] OR "totaled"[All Fields] OR "totaling"[All Fields] OR "totalled"[All Fields] OR "totalling"[All Fields] OR "totals"[All Fields]*

occlusion: *"dental occlusion"[MeSH Terms] OR ("dental"[All Fields] AND "occlusion"[All Fields]) OR "dental occlusion"[All Fields] OR "occlusion"[All Fields] OR "occlused"[All Fields] OR "occlusions"[All Fields] OR "occlusive"[All Fields] OR "occlusives"[All Fields]*

CTO: *"GMS Curr Top Otorhinolaryngol Head Neck Surg"[Journal:__jid101275966] OR "Cells Tissues Organs"[Journal:__jid100883360] OR "Cardiovasc Thorac Open"[Journal:__jid101735643] OR "cto"[All Fields]*

chronic: *"chronic"[All Fields] OR "chronical"[All Fields] OR "chronically"[All Fields] OR "chronicities"[All Fields] OR "chronicity"[All Fields] OR "chronicization"[All Fields] OR "chronics"[All Fields]*

coronary occlusion: *"coronary occlusion"[MeSH Terms] OR ("coronary"[All Fields] AND "occlusion"[All Fields]) OR "coronary occlusion"[All Fields]*

percutaneous coronary intervention: *"percutaneous coronary intervention"[MeSH Terms] OR ("percutaneous"[All Fields] AND "coronary"[All Fields] AND "intervention"[All Fields]) OR "percutaneous coronary intervention"[All Fields]*

optimal: *"optimal"[All Fields] OR "optimality"[All Fields] OR "optimally"[All Fields] OR "optimization"[All Fields] OR "optimizations"[All Fields] OR "optimize"[All Fields] OR "optimized"[All Fields] OR "optimizer"[All Fields] OR "optimizers"[All Fields] OR "optimizes"[All Fields] OR "optimizing"[All Fields]*

medical: *"medic"[All Fields] OR "medical"[All Fields] OR "medicalization"[MeSH Terms] OR "medicalization"[All Fields] OR "medicalizations"[All Fields] OR "medicalize"[All Fields] OR "medicalized"[All Fields] OR "medicalizes"[All Fields] OR "medicalizing"[All Fields] OR "medically"[All Fields] OR "medicals"[All Fields] OR "medicated"[All Fields] OR "medication's"[All Fields] OR "medics"[All Fields] OR "pharmaceutical preparations"[MeSH Terms] OR ("pharmaceutical"[All Fields] AND "preparations"[All Fields]) OR "pharmaceutical preparations"[All Fields] OR "medication"[All Fields] OR "medications"[All Fields]*

treatment: *"therapeutics"[MeSH Terms] OR "therapeutics"[All Fields] OR "treatments"[All Fields] OR "therapy"[Subheading] OR "therapy"[All Fields] OR "treatment"[All Fields] OR "treatment's"[All Fields]*

conservative treatment: *"conservative treatment"[MeSH Terms] OR ("conservative"[All Fields] AND "treatment"[All Fields]) OR "conservative treatment"[All Fields]*

Web of Science

(<chronic total occlusion> OR <CTO> OR <chronic coronary occlusion>) AND (<percutaneous coronary intervention> OR <PCI>) AND (<optimal medical treatment> OR <conservative treatment>)

Cochrane Library

(<chronic total occlusion> OR <CTO> OR <chronic coronary occlusion>) AND (<percutaneous coronary intervention> OR <PCI>) AND (<optimal medical treatment> OR <conservative treatment>) in Title Abstract Keyword

Figure S1: Funnel plot of primary outcome analysis

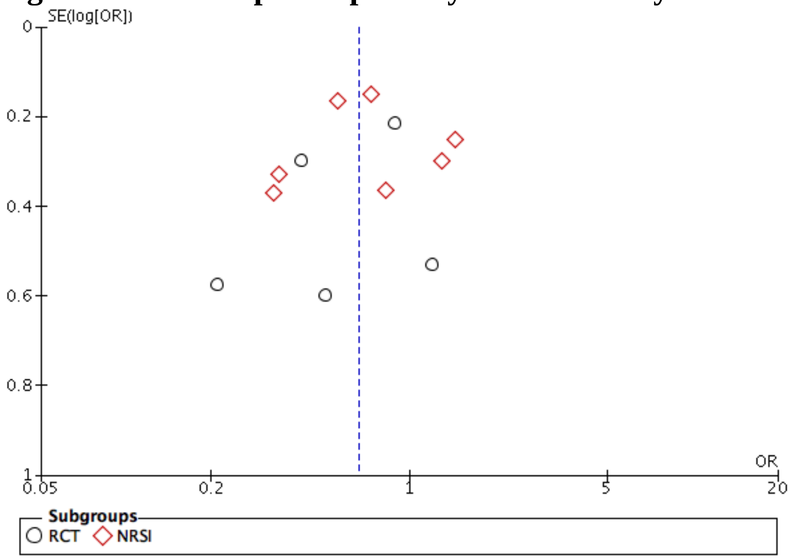

Figure S2: Mortality&MI

Study or Subgroup	PCI Events	PCI Total	OMT Events	OMT Total	Weight	Odds Ratio M-H, Random, 95% CI
**9.1.1 RCT**						
COMET CTO	3	50	7	50	2.8%	0.39 [0.10, 1.61]
DECISION	15	417	21	398	9.6%	0.67 [0.34, 1.32]
EURO-CTO	16	259	5	137	4.9%	1.74 [0.62, 4.85]
EXPLORE	6	148	2	154	2.1%	3.21 [0.64, 16.17]
REVASC	1	101	3	104	1.1%	0.34 [0.03, 3.29]
**Subtotal (95% CI)**		**975**		**843**	**20.5%**	**0.92 [0.45, 1.87]**
Total events	41		38			
Heterogeneity: Tau² = 0.26; Chi² = 6.72, df = 4 (P = 0.15); I² = 40%						
Test for overall effect: Z = 0.24 (P = 0.81)						
**9.1.2 NRSI**						
CTO KUGH registry	11	265	23	265	8.4%	0.46 [0.22, 0.95]
Gong 2021	10	300	21	263	7.9%	0.40 [0.18, 0.86]
Guo 2021	39	402	68	402	17.6%	0.53 [0.35, 0.80]
Qin 2022	8	108	15	108	6.1%	0.50 [0.20, 1.22]
SAMSUNG CTO Registry	72	456	111	456	21.9%	0.58 [0.42, 0.81]
Wu 2022	15	128	29	117	9.5%	0.40 [0.20, 0.80]
Yan 2020	16	118	15	118	8.1%	1.08 [0.51, 2.29]
**Subtotal (95% CI)**		**1777**		**1729**	**79.5%**	**0.55 [0.44, 0.67]**
Total events	171		282			
Heterogeneity: Tau² = 0.00; Chi² = 4.97, df = 6 (P = 0.55); I² = 0%						
Test for overall effect: Z = 5.76 (P < 0.00001)						
**Total (95% CI)**		**2752**		**2572**	**100.0%**	**0.60 [0.47, 0.76]**
Total events	212		320			
Heterogeneity: Tau² = 0.04; Chi² = 14.70, df = 11 (P = 0.20); I² = 25%						
Test for overall effect: Z = 4.14 (P < 0.0001)						
Test for subgroup differences: Chi² = 1.87, df = 1 (P = 0.17), I² = 46.5%						

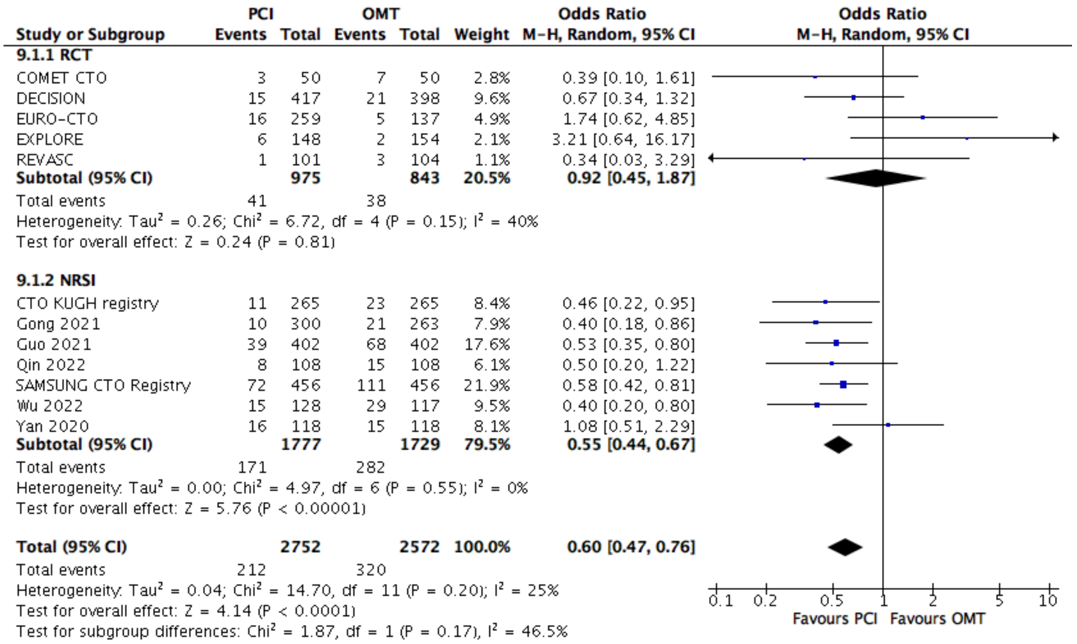

Figure S3: Stroke

Study or Subgroup	PCI Events	PCI Total	OMT Events	OMT Total	Weight	Odds Ratio M-H, Random, 95% CI
**7.1.1 RCT**						
COMET CTO	0	50	0	50		Not estimable
DECISION	6	417	10	398	34.4%	0.57 [0.20, 1.57]
EURO-CTO	5	259	1	137	7.7%	2.68 [0.31, 23.15]
EXPLORE	0	148	2	154	3.9%	0.21 [0.01, 4.31]
**Subtotal (95% CI)**		**874**		**739**	**46.0%**	**0.70 [0.25, 1.97]**
Total events	11		13			
Heterogeneity: Tau² = 0.14; Chi² = 2.27, df = 2 (P = 0.32); I² = 12%						
Test for overall effect: Z = 0.68 (P = 0.50)						
**7.1.2 NRSI**						
Budrys 2021	1	163	2	215	6.2%	0.66 [0.06, 7.31]
CTO KUGH registry	3	265	3	265	13.9%	1.00 [0.20, 5.00]
Gong 2021	1	300	3	263	7.0%	0.29 [0.03, 2.80]
IRCTO	1	619	3	619	7.0%	0.33 [0.03, 3.20]
Qin 2022	1	108	6	108	7.9%	0.16 [0.02, 1.34]
Wu 2022	2	128	4	117	12.2%	0.45 [0.08, 2.49]
**Subtotal (95% CI)**		**1583**		**1587**	**54.0%**	**0.45 [0.20, 1.02]**
Total events	9		21			
Heterogeneity: Tau² = 0.00; Chi² = 2.19, df = 5 (P = 0.82); I² = 0%						
Test for overall effect: Z = 1.92 (P = 0.05)						
**Total (95% CI)**		**2457**		**2326**	**100.0%**	**0.54 [0.30, 0.99]**
Total events	20		34			
Heterogeneity: Tau² = 0.00; Chi² = 4.88, df = 8 (P = 0.77); I² = 0%						
Test for overall effect: Z = 2.00 (P = 0.05)						
Test for subgroup differences: Chi² = 0.43, df = 1 (P = 0.51), I² = 0%						

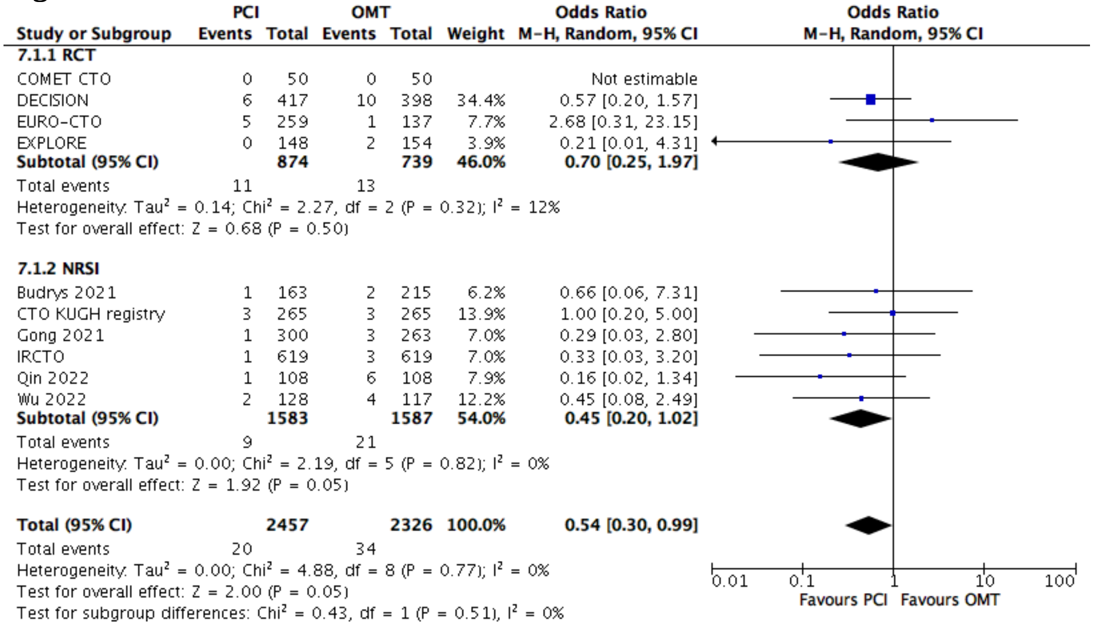

Figure S4: Major adverse cardiac and cerebrovascular events

Study or Subgroup	PCI Events	PCI Total	OMT Events	OMT Total	Weight	Odds Ratio M-H, Random, 95% CI
**8.1.1 RCT**						
COMET CTO	5	50	9	50	7.1%	0.51 [0.16, 1.63]
DECISION	54	417	61	398	16.5%	0.82 [0.55, 1.22]
EURO-CTO	30	259	29	137	14.1%	0.49 [0.28, 0.85]
EXPLORE	8	148	9	154	8.8%	0.92 [0.35, 2.45]
**Subtotal (95% CI)**		**874**		**739**	**46.6%**	**0.70 [0.52, 0.94]**
Total events	97		108			
Heterogeneity: Tau² = 0.00; Chi² = 2.83, df = 3 (P = 0.42); I² = 0%						
Test for overall effect: Z = 2.40 (P = 0.02)						
**8.1.2 NRSI**						
CTO KUGH registry	47	265	35	265	15.4%	1.42 [0.88, 2.28]
Gong 2021	12	300	30	263	12.3%	0.32 [0.16, 0.65]
Qin 2022	18	108	26	108	12.5%	0.63 [0.32, 1.23]
Wu 2022	19	128	40	117	13.3%	0.34 [0.18, 0.62]
**Subtotal (95% CI)**		**801**		**753**	**53.4%**	**0.57 [0.27, 1.21]**
Total events	96		131			
Heterogeneity: Tau² = 0.50; Chi² = 18.62, df = 3 (P = 0.0003); I² = 84%						
Test for overall effect: Z = 1.46 (P = 0.15)						
**Total (95% CI)**		**1675**		**1492**	**100.0%**	**0.62 [0.42, 0.92]**
Total events	193		239			
Heterogeneity: Tau² = 0.20; Chi² = 21.49, df = 7 (P = 0.003); I² = 67%						
Test for overall effect: Z = 2.39 (P = 0.02)						
Test for subgroup differences: Chi² = 0.23, df = 1 (P = 0.63), I² = 0%						

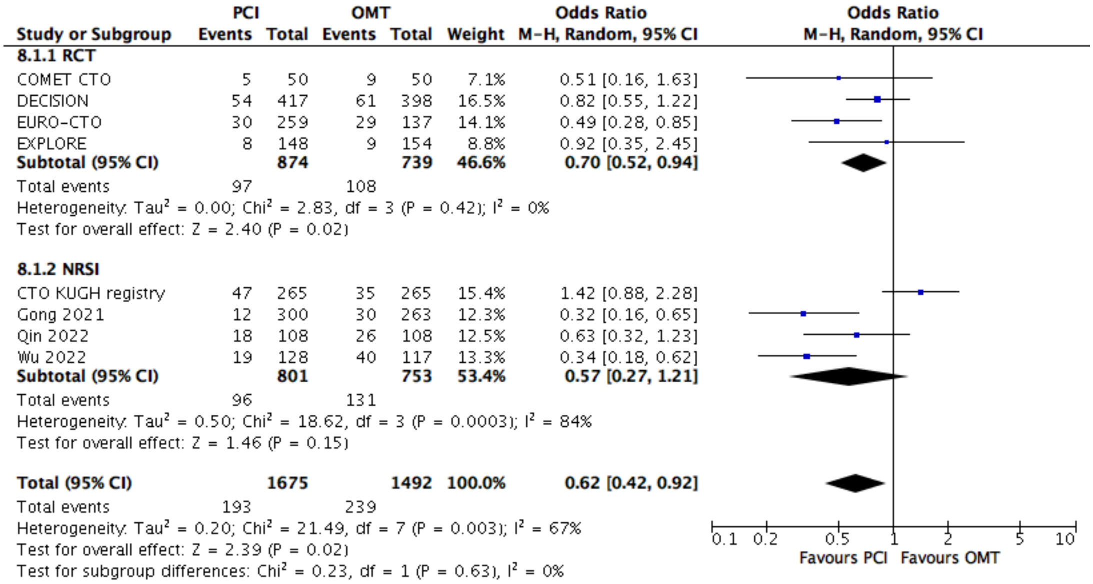

Figure S5: Multipaneled figure of sensitivity and subgroup analysis

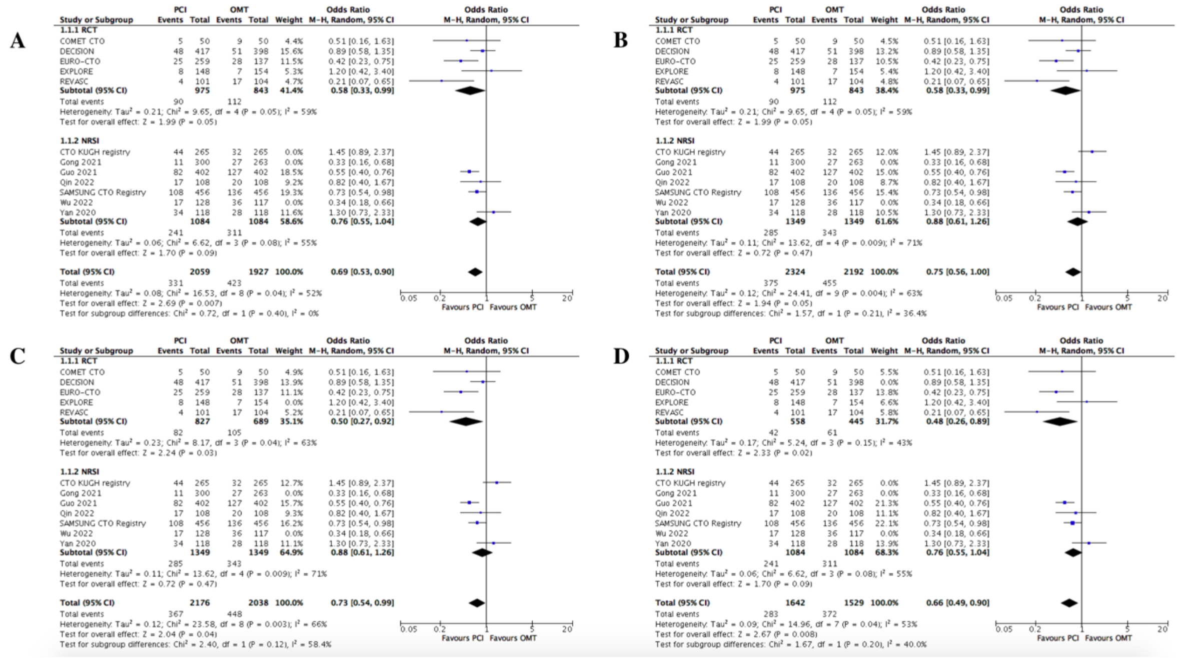

Table S1: Event data on efficacy outcomes

	Treatment	All cause death	Cardiac death	Myocardial infarction	Target lesion revascularization	Target vessel revascularization	Stroke	Major adverse cardiac events	Major adverse cardiac and cerebrovascular events
**Randomized-controlled trials**									
**EXPLORE**	PCI	-	4/148	2/148	-	2/148	0/148	8/148	8/148
	OMT	-	0/154	2/154	-	5/154	2/154	7/154	9/154
**REVASC**	PCI	1/101	1/101	0/101	-	3/101	-	4/101	-
	OMT	2/104	2/104	1/104	-	14/104	-	17/104	-
**IMPACTOR**	PCI	0/39	0/39	-	-	2/39	-	-	-
	OMT	0/33	0/33	-	-	0/33	-	-	-
**DECISION**	PCI	15/417	8/417	7/417	-	33/417	6/417	48/417	54/417
	OMT	21/398	14/398	7/398	-	30/398	10/398	51/398	61/398
**EURO-CTO**	PCI	16/259	8/259	8/259	9/259	9/259	5/259	25/259	30/259
	OMT	5/137	3/137	2/137	23/137	23/137	1/137	28/137	29/137
**COMET CTO**	PCI	4/50	3/50	0/50	-	2/50	0/50	5/50	5/50
	OMT	11/50	6/50	1/50	-	2/50	0/50	9/50	9/50
**Non-randomized controlled studies of intervention**									
**Álvarez-Contreras 2021**	PCI	34/240	14/240	18/240	-	-	-	-	-
	OMT	301/719	185/719	57/719	-	-	-	-	-
**Budrys 2021**	PCI	17/163	-	11/163	-	2/163	1/163	-	-
	OMT	41/215	-	13/215	-	3/215	2/215	-	-
**CTO KUGH registry 2018**	PCI	11/265	7/265	4/265	28/265	33/265	3/265	44/265	47/265
	OMT	17/265	10/265	13/265	8/265	9/265	3/265	32/265	35/265
**Choo 2019**	PCI	27/264	-	-	-	-	-	-	-
	OMT	28/264	-	-	-	-	-	-	-
**Gong 2021**	PCI	7/300	4/300	6/300	-	1/300	1/300	11/300	12/300
	OMT	17/263	16/263	5/263	-	6/263	3/263	27/263	30/263
**Guo 2021**	PCI	-	10/402	29/402	-	43/402	-	82/402	-
	OMT	-	20/402	48/402	-	59/402	-	127/402	-
**Kook 2021**	PCI	96/1341	47/1341	18/1341	-	-	-	-	-
	OMT	143/832	88/832	19/832	-	-	-	-	-
**Ladwiniec 2015**	PCI	34/294	14/294	7/294	-	-	-	-	-
	OMT	49/294	19/294	8/294	-	-	-	-	-
**SAMSUNG CTO registry**	PCI	101/456	48/456	24/456	36/456	36/456	-	108/456	-
	OMT	140/456	71/456	40/456	25/456	25/456	-	136/456	-
**IRCTO**	PCI	-	9/619	7/619	-	-	1/619	-	-
	OMT	-	27/619	18/619	-	-	3/619	-	-
**Qin 2022**	PCI	6/108	4/108	4/108	-	9/108	1/108	17/108	18/108
	OMT	14/108	8/108	7/108	-	5/108	6/108	20/108	26/108
**Wu 2022**	PCI	17/128	11/128	4/128	2/128	2/128	2/128	17/128	19/128
	OMT	28/117	21/117	8/117	7/117	7/117	4/117	36/117	40/117
**Yan 2020**	PCI	10/118	6/118	10/118	-	18/118	-	34/118	-
	OMT	13/118	11/118	4/118	-	13/118	-	28/118	-
